# None of the Rotor Residues of F_1_-ATPase Are Essential for Torque Generation

**DOI:** 10.1016/j.bpj.2014.04.013

**Published:** 2014-05-20

**Authors:** Ryohei Chiwata, Ayako Kohori, Tomonari Kawakami, Katsuyuki Shiroguchi, Shou Furuike, Kengo Adachi, Kazuo Sutoh, Masasuke Yoshida, Kazuhiko Kinosita

**Affiliations:** † Department of Physics, Faculty of Science and Engineering, Waseda University, Shinjuku-ku, Tokyo, Japan; ‡ ATP Synthesis Regulation Project, ICORP, Japan Science and Technology Agency (JST), Aomi 2-41, Koto-ku, Tokyo, Japan; § Department of Molecular Bioscience, Kyoto Sangyo University, Motoyama, Kamigamo, Kyoto, Japan

## Abstract

F_1_-ATPase is a powerful rotary molecular motor that can rotate an object several hundred times as large as the motor itself against the viscous friction of water. Forced reverse rotation has been shown to lead to ATP synthesis, implying that the mechanical work against the motor’s high torque can be converted into the chemical energy of ATP. The minimal composition of the motor protein is *α*_3_*β*_3_*γ* subunits, where the central rotor subunit *γ* turns inside a stator cylinder made of alternately arranged *α*_3_*β*_3_ subunits using the energy derived from ATP hydrolysis. The rotor consists of an axle, a coiled coil of the amino- and carboxyl-terminal *α*-helices of *γ*, which deeply penetrates the stator cylinder, and a globular protrusion that juts out from the stator. Previous work has shown that, for a thermophilic F_1_, significant portions of the axle can be truncated and the motor still rotates a submicron sized bead duplex, indicating generation of up to half the wild-type (WT) torque. Here, we inquire if any specific interactions between the stator and the rest of the rotor are needed for the generation of a sizable torque. We truncated the protruding portion of the rotor and replaced part of the remaining axle residues such that every residue of the rotor has been deleted or replaced in this or previous truncation mutants. This protrusionless construct showed an unloaded rotary speed about a quarter of the WT, and generated one-third to one-half of the WT torque. No residue-specific interactions are needed for this much performance. F_1_ is so designed that the basic rotor-stator interactions for torque generation and control of catalysis rely solely upon the shape and size of the rotor at very low resolution. Additional tailored interactions augment the torque to allow ATP synthesis under physiological conditions.

## Introduction

F_1_-ATPase is a rotary molecular motor in which the central *γ* subunit rotates inside a stator cylinder made of alternately arranged *α*_3_*β*_3_ subunits (1–7). The torque that drives *γ* derives from ATP hydrolysis in the three catalytic sites residing at *β*-*α* interfaces, primarily hosted by a *β* subunit (8). In crystal structures, mostly of mitochondrial F_1_ or MF_1_ (7–9), the *β* subunit binding a nucleotide and an empty *β* adopt quite different conformations, whereas variations in *α* structures are less conspicuous, leading to the proposal that nucleotide-dependent bending and unbending of *β* confers torque to γ (10). Stepwise conformational changes in *β*, including one so far unseen in crystals, have indeed been detected during active rotation (11). Although the free energy drop in the overall ATP hydrolysis reaction drives *γ* rotation, the *γ* rotor in turn coordinates the progress of sequential hydrolysis in the three catalytic sites by specifying which of the chemical reaction steps (ATP binding, cleavage of ATP, and product releases) are to proceed at a particular rotary angle (12,13). The *γ*-dictated catalysis is best illustrated by the experiments where reverse rotation of *γ* led to ATP synthesis, i.e., reversal of the hydrolysis reaction in the catalytic sites (14,15). The *γ* rotation and the control of catalysis must both be mediated by rotor-stator interactions, which would involve specific contacts between key residues such as the hydrogen-bonding “catch” interactions (8).

**Figure 1:**
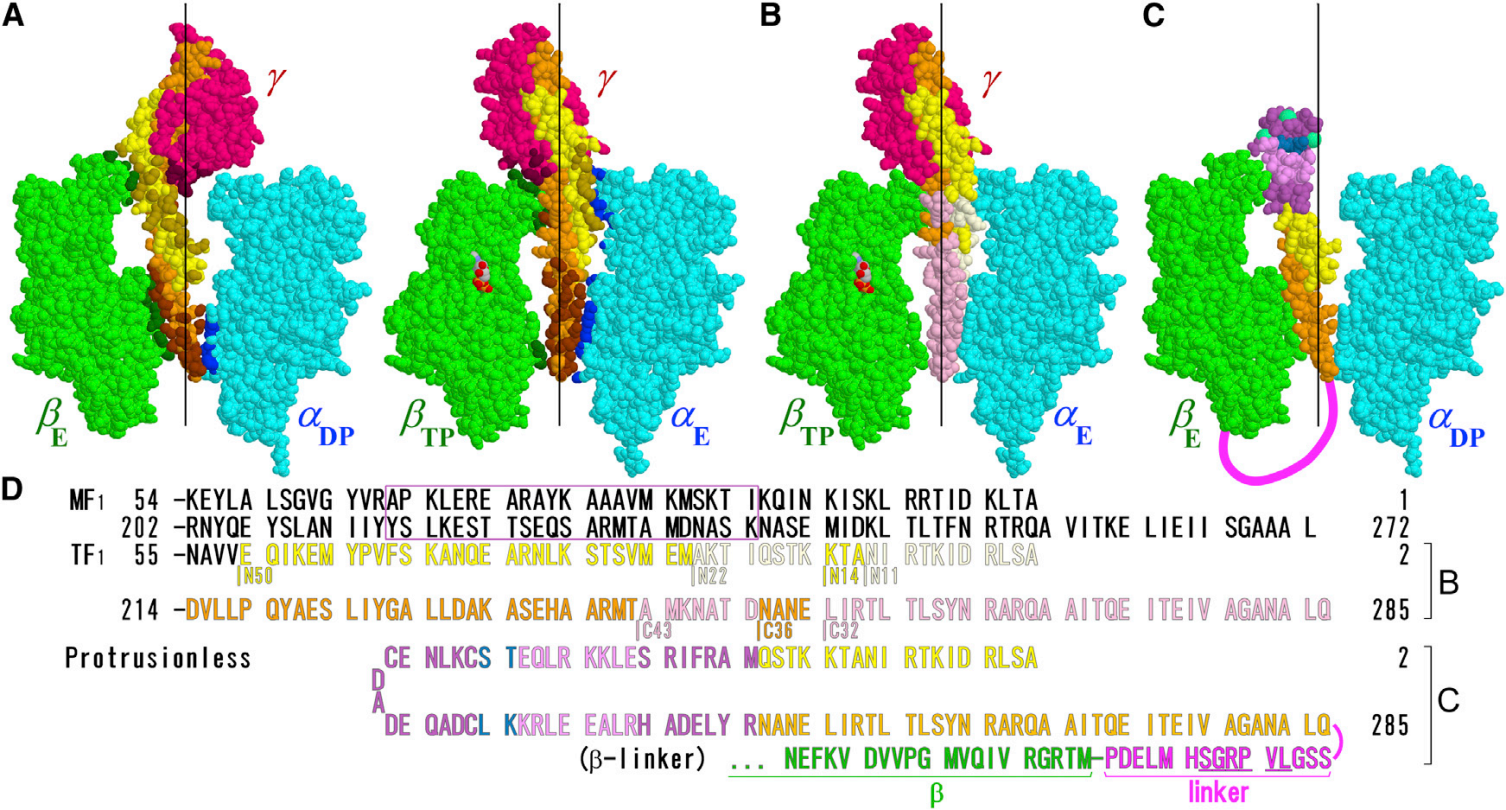
Design of rotor mutations based on a crystal structure of MF_1_ (9). (*A*) Rotor-stator arrangement. The *γ* rotor (*dark pink*) and an opposing pair of a *β* subunit (*green*) and an *α* subunit (*cyan*) are shown for two views differing by 120°. The *α* and *β* subunits are designated according to the nucleotide bound in the original structure (8): *TP*, ATP (analog); *DP*, ADP; *E*, empty. The C-terminal *α*-helix of *γ* is painted orange, and N-terminal helix yellow. Dark-colored atoms in *β* and *α* are those within 0.5 nm of an atom in *γ* (*excluding hydrogen*), and dark atoms in *γ* are within 0.5 nm of *β* or *α*. The bound nucleotide is in CPK color. Black vertical lines show a putative rotation axis (10). (*B*) Positions of previous rotor truncations as diagrammed in *D*. (*C*) The protrusionless mutant. The exogenous hairpin is borrowed from Rop (*dark violet*) and seryl-tRNA synthetase (*light violet*) with extra four residues (*blue*) as diagramed in *D*. The hairpin structure shown is part of the MF_1_ structure enclosed in the violet rectangle in *D* (actual structure and position of the modified *γ* are unknown). Sea-green spheres show approximate positions of the three cysteines. Thick magenta line represents the linker between *β* and *γ*. The other two *γ*s must be outside the stator cylinder. (*D*) N- and C-terminal sequences of the *γ* subunit of MF_1_ and TF_1_, showing the positions of truncations/replacements and the linker (*thrombin site underlined*) sequence for *C*. The sequence for TF_1_ starts from 2, because we count from the first Met, which is processed in the WT and this protrusionless construct but not in some mutants (18). To see this figure in color, go online.

Studies of thermophilic F_1_-ATPase (TF_1_), however, have shown that a large part of the rotor subunit is dispensable for rotation and ATP hydrolysis. The *γ* axle that deeply penetrates the stator cylinder comprises coiled coil of an amino (N-) and a carboxyl (C-) terminal *α*-helix (Fig. 1 *A*). Truncation up to 36 residues of the C-terminal helix (ΔC36 in Fig. 1, *B* and *D*; (16)) or of the entire 50 residues of the N-terminal helix (ΔN50 in Fig. 1 *B*; (17)) still retained a sizable torque that allowed rotation of a duplex of submicron beads. When both helices were cut level, ΔN11C32 could rotate the bead duplex but ΔN14C36 could not (18). The “axle-less” mutant (ΔN22C43 in Fig. 1 *B*) was barely able to rotate a bead of negligible load (a 40-nm gold bead), albeit at a very low speed of ∼1 revolution per second (rps) compared to ∼200 rps of wild-type (WT) (18). More recently, a rotorless construct (*α*_3_*β*_3_ alone) has been shown to undergo circular conformational changes in the same direction as the *γ* rotation (19), indicating that the hydrolysis reactions in the three catalytic sites are coordinated without the help of the central *γ*. The circular conformational changes, however, were slow and of similar speed to the axle-less mutant. The implication is that the stator alone (*α*_3_*β*_3_) has the potential of rotating an object that fits in the central groove, such as the axle-less rotor, but the rotation would be extremely slow and powerless unless proper rotor-stator interactions are provided. ATP synthesis should require a high torque, because the mechanical work done against the motor torque is the source of energy needed for the synthesis.

Here, we inquire whether any specific rotor-stator interaction is needed for rotation and finite torque generation. To supplement the previous studies where the axle portion of the rotor was truncated, we deleted the entire protruding portion, leaving only part of the N- and C-terminal helices. We joined the remaining two short helices with a cap sequence borrowed from coiled coils of unrelated proteins, hoping that the resulting antiparallel coiled coil would serve as a rotor (Fig. 1 *C*). This protrusionless construct rotated at an unloaded speed ∼1/4 of the WT and with a torque 1/3∼1/2 of the WT. All intrinsic residues of the rotor of TF_1_ have been removed in this or previous constructs, implying that no residue-specific interaction is required for the generation of a sizable torque.

## Materials and Methods

### Molecular genetics

The TF_1_ that we regard as the WT in this work is a subcomplex consisting of *α*_3_*β*_3_*γ* subunits. It is derived from the thermophile *Bacillus* PS3 and contains the following mutations: *α*-(C193S), *β*-(His_10_ at N-terminus), and *γ*-(S109C, I212C) in the revised TF_1_ sequence (17,20). Starting with plasmid pKABG1/HC95 that carries genes for the WT subcomplex, we previously constructed a *β*-*γ* fusion mutant where we connected the C-terminus of *γ* and the N-terminus of *β* with a 15-residue linker including a thrombin site (Fig. 1 *D*) and, instead of the *β*-His_10_ removed for the connection, we added a His_6_ tag at the N-terminus of *α*. In this work, we started with the *β*-*γ* fusion mutant and replaced its residues *γ*20–249 with a 48-residue hairpin sequence shown in Fig. 1 *D*. The middle of the replacement sequence is a helix-turn-helix motif (residues 24–37 in Protein Data Base (PDB) code 1rop) of an RNA-binding protein Rop that forms an antiparallel coiled coil (21). This motif that would form a cap of the truncated *γ* is flanked by two exogenous residues on each side, and then by sequences in an antiparallel coiled-coil region of seryl-tRNA synthetase (residues 39–46 and 81–88 in PDB code 1set; (22)) and another coiled-coil pair from Rop (residues 11–17 and 44–50). This complex chimeric design is due to our step-by-step attempts at eliminating all TF_1_ residues that we have not deleted in previous studies. Near the hairpin cap we introduced three cysteines for bead attachment. The plasmid for the *β*-*γ* fusion mutant contained Bgl II_1732_ and Mlu I_2783_ sites that encompass the entire *γ* sequence. Thus, we synthesized the designed DNA sequence between these sites (gene synthesis service, Eurofins MWG Operon, Ebersberg, Germany) and introduced it into the *β*-*γ* fusion mutant. We sequenced the final expression plasmid for confirmation.

### Purification of mutant TF_1_

WT and protrusionless TF_1_ were expressed in *Escherichia coli* strain JM103 Δ(*uncB*-*uncD*), purified and biotinylated basically as described (17,23) but without the butyl column treatment for removal of bound nucleotides. Because the mutant tended to accumulate in inclusion bodies, we terminated *E. coli* culture at 10 h for the mutant instead of the usual 12–15 h for the WT. The cells were suspended in buffer A (50 mM imidazole, pH 7.0, 100 mM potassium phosphate, 100 mM KCl) and sonicated for 12 min on ice. After centrifugation, we heated the supernatant at 65°C for 5 min, shorter than the usual 15 min, and centrifuged. The supernatant was applied to a Ni^2+^-nitrilotriacetic acid (Ni-NTA) column (Qiagen, Hilden, Germany) and washed with buffer A. F_1_ complex was eluted with 300 mM imidazole (pH 7.0) and stored as ammonium sulfate precipitate at 4°C. The precipitate was resuspended in buffer B (10 mM MOPS, 100 mM KCl, pH 7.0) containing 5 mM DTT and, after 40–60 min, passed through a size-exclusion column (Superdex 200 10/300 GL, GE Healthcare, Uppsala, Sweden) equilibrated with buffer B. The WT produced a prominent peak for the assembled *α*_3_*β*_3_*γ* complex, but the protrusionless mutant showed, in addition, an early peak for apparent aggregates and a lagging peak likely representing *α* monomers, both of which overlapped with the peak corresponding to the *α*_3_(*β*-*γ*)_3_ complex. The extra *γ* subunits that cannot be accommodated inside the stator cylinder seem to be responsible for the aggregation, because the aggregate peak was significantly reduced by thrombin treatment. The aggregates and monomers mostly disappeared after the second passage after biotinylation below. The *β*-*γ* fusion mutant without *γ* deletion was purified as described (17).

For observation of rotation, we biotinylated cysteines on *γ* by incubation with a fourfold molar excess over F_1_ of biotin-PEAC_5_-maleimide (Dojindo, Kumamoto, Japan) in buffer C (10 mM MOPS, 50 mM KCl, 4 mM MgCl_2_, pH 7.0) for 30 min at room temperature. Unreacted biotin was removed on the size-exclusion column with buffer B, and we collected the peak fraction(s) corresponding to the assembled complex.

Cleavage of the *β*-*γ* link, when attempted, was done simultaneously with the biotinylation by adding 4 NIH units of thrombin (bovine plasma, Sigma Aldrich, St. Louis, MO) per nmol F_1_ in the last 10 min of the 30-min incubation.

The concentration of F_1_ was determined from the absorbance using *ε*_280nm_ of 154,000 M^−1^ cm^−1^. This leads to a slight underestimate of the concentration of the protrusionless mutant, which we ignore.

### Measurement of hydrolysis activity

The rate of ATP hydrolysis at 2 mM ATP (Roche Diagnostics, Manheim, Germany) and 4 mM Mg^2+^ was measured at 23°C by coupling the hydrolysis reaction with NADH consumption, as described (16). We added biotinylated F_1_ at a final concentration of 2 nM into an assay mixture consisting of 2 mM ATP, 0.2 mM NADH, 1 mM phosphoenolpyruvate, 250 *μ*g mL^−1^ pyruvate kinase (rabbit muscle, Roche) and 50 *μ*g mL^−1^ lactate dehydrogenase (hog muscle, Roche) in buffer C. Because we did not remove bound nucleotides completely, we also added 0.3% (w/v) LDAO (Lauryldimethylamine *N*-oxide, Sigma) to relieve F_1_ of MgADP inhibition. Hydrolysis proceeded at a constant rate in the presence of LDAO, and we estimated the rate in a 50-s portion.

### Observation of rotation

The observation chamber and the imaging system have been described (17). Briefly, the bottom of an observation chamber was a thiol-silanized coverslip functionalized with Ni-NTA and the top was an untreated coverslip. For rotation of 40-nm gold beads, we infused 1 nM of biotinylated F_1_ in buffer D (10 mM MOPS, 50 mM KCl, pH 7.0), and then 5 mg mL^−1^ BSA in buffer D, gold beads (EM.GC40, BBInternational, Cardiff, UK) coated with polyethylene glycol and functionalized with streptavidin, and finally buffer C with the addition of 2 mM ATP and an ATP regeneration system (0.2 mg mL^−1^ creatine kinase (rabbit muscle, Roche) and 2.5 mM creatine phosphate (Nacalai Tesque, Kyoto, Japan)). Rotation was observed at 23°C by laser dark-field microscopy (18) on an inverted microscope (IX70, Olympus, Tokyo, Japan). Images were captured with a high-speed CMOS camera (FASTCAM-F1-DJV, Photron, Tokyo, Japan) at 4000–8000 frames s^−1^ as an 8-bit AVI file. Rotation of polystyrene beads was observed similarly by infusing 0.1% (w/v) of streptavidin-coated beads of diameter 0.29 *μ*m (Thermo Scientific, Indianapolis, IN) or 0.49 *μ*m (Bangs Laboratories, Fishers, IN). The applied F_1_ concentration was 5 nM for the protrusionless mutant and 1 nM for the WT and the *β*-*γ* fusion mutant. Bright-field images were recorded at 500 frames s^−1^ by the above camera. The number of rotating polystyrene beads was counted in randomly chosen 18 fields of view (31 × 31 *μ*m^2^ each) per chamber for the total of six chambers.

The torque *Ν* the motor generated was calculated from the angular velocity *ω* in rad s^−1^ of a 0.29- or 0.49-*μ*m bead duplex as (24,25)

(1) N=ωξ,

where ***ξ*** is the frictional drag coefficient given, for the case of a duplex of spherical beads, by

(2) $$\xi =2\times 8\pi \eta a^{3}+6\pi \eta ax_{1}^{2}+6\pi \eta ax_{2}^{2},$$

where *a* is the bead radius, *x*_1_ and *x*_2_ the radii of the revolution of the inner and outer beads, and *η* is the viscosity of the medium (0.93 × 10^−3^ N s m^−2^ at 23°C). We selected those duplexes with *x*_2_ > 200 nm for 0.29-*μ*m beads and *x*_2_ > 400 nm for 0.49-*μ*m beads; *x*_1_ was taken as 0. The drag above is likely an underestimate (24,26).

## Results

### The protrusionless TF_1_

Previous studies have shown that, of all the rotor residues of TF_1_, the N-terminal 50 residues (*γ*2–51 in TF_1_ sequence; Fig. 1 *D*) and the C-terminal 36 residues (*γ*250–285) are dispensable for finite torque generation. In this work, we started with a mutated TF_1_ subcomplex *α*(C193S)_3_*β*(His_10_ at N-terminus)_3_*γ*(S109C, I212C) that we regard as the WT for rotation and catalysis assays, and removed residues *γ*20–249 to see if there are indispensable residues in the middle part of the rotor. The remaining N- and C-termini of *γ* were joined with a 48-residue hairpin sequence (Fig. 1 *D*) designed as continuous patches of antiparallel coiled coils of a Rop protein (21) and seryl-tRNA synthetase (22). If the joined chain forms a coiled coil as expected, its gross structure will mimic the coiled-coil axle of the WT *γ* (Fig. 1 *C*), but no residues in the introduced hairpin match the original sequence except for *γ*-Leu-32, which has already been shown to be dispensable.

**Figure 2:**
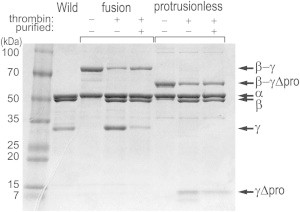
Confirmation of mutation by SDS-PAGE. Samples were run in a 10–20% gradient gel, and stained with Coomasie Brilliant Blue R-250. The *β*-*γ* fusion mutant and the protrusionless mutant were each run in three lanes, where – – indicates purified subcomplex before thrombin treatment, + – after thrombin treatment for 10 min (*reaction mixture*), and + + the subcomplex after further purification by size-exclusion chromatography. *γ*Δpro, the *γ* subunit of the protrusionless mutant.

To assist the assembly of the rotor-stator complex, we genetically connected the C-terminus of the protrusionless *γ* to the *β* N-terminus via a flexible peptide linker including a thrombin site, as done previously ((17); Fig. 1 *D*). Instead of the *β* His_10_ tag that we eliminated for linking, we introduced His_6_ tag at the N-terminus of *α*. The assembled complex would contain three modified *γ* (each connected to a *β*), of which only one could occupy the central hole of the stator. As in the previous study, treatment with thrombin left part of the *β*-**γ** linkage intact (Fig. 2), suggesting that the thrombin site would be occluded when the associated *γ* is inserted into the stator. The previous study also showed that the extra *γ*s outside the stator do not impair rotation and hydrolysis rates significantly, which turned out to be the case for the protrusionless mutant as well (see below). At the tip of the hairpin, which would protrude from the stator, we introduced three cysteine residues for probe attachment (anticipated positions shown in *sea green* in Fig. 1 *C*). These are the sole cysteines in the complex.

For the observation of rotation, we biotinylated the cysteines and attached either a 40-nm gold bead or a duplex of submicron polystyrene beads, both modified with streptavidin. We did not confirm which of the three cysteines participated in bead attachment. The protrusionless complex was attached to a glass surface via the His_6_ tags on the three *α* subunits. For comparison, we also observed the rotation of the *β*-*γ* fusion mutant without a *γ* modification (*β*-*γ*ΔN0 in (17)), also attached via His_6_ tags on *α*, and of the WT attached via His_10_ tags on *β*.

### Unloaded rotation and ATP hydrolysis activity

**Figure 3:**
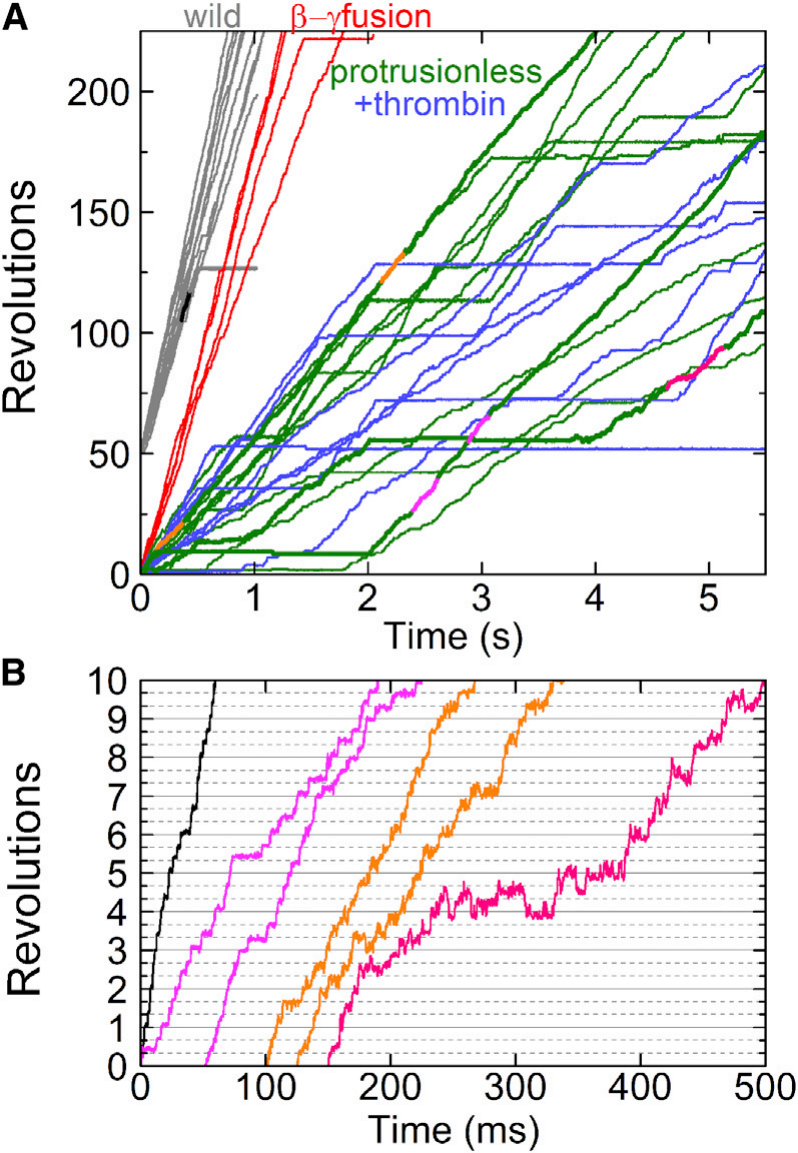
Time courses of 40-nm gold bead rotation recorded at 4000 or 8000 frames s^−1^. (*A*) Overall time courses. Portions shown in a different color are magnified in *B* with the same color. (*B*) Magnified time courses corresponding to portions in *A* shown in the same color. Horizontal solid lines are spaced every one revolution, and gray dotted lines every 120°. To see this figure in color, go online.

**Figure 4:**
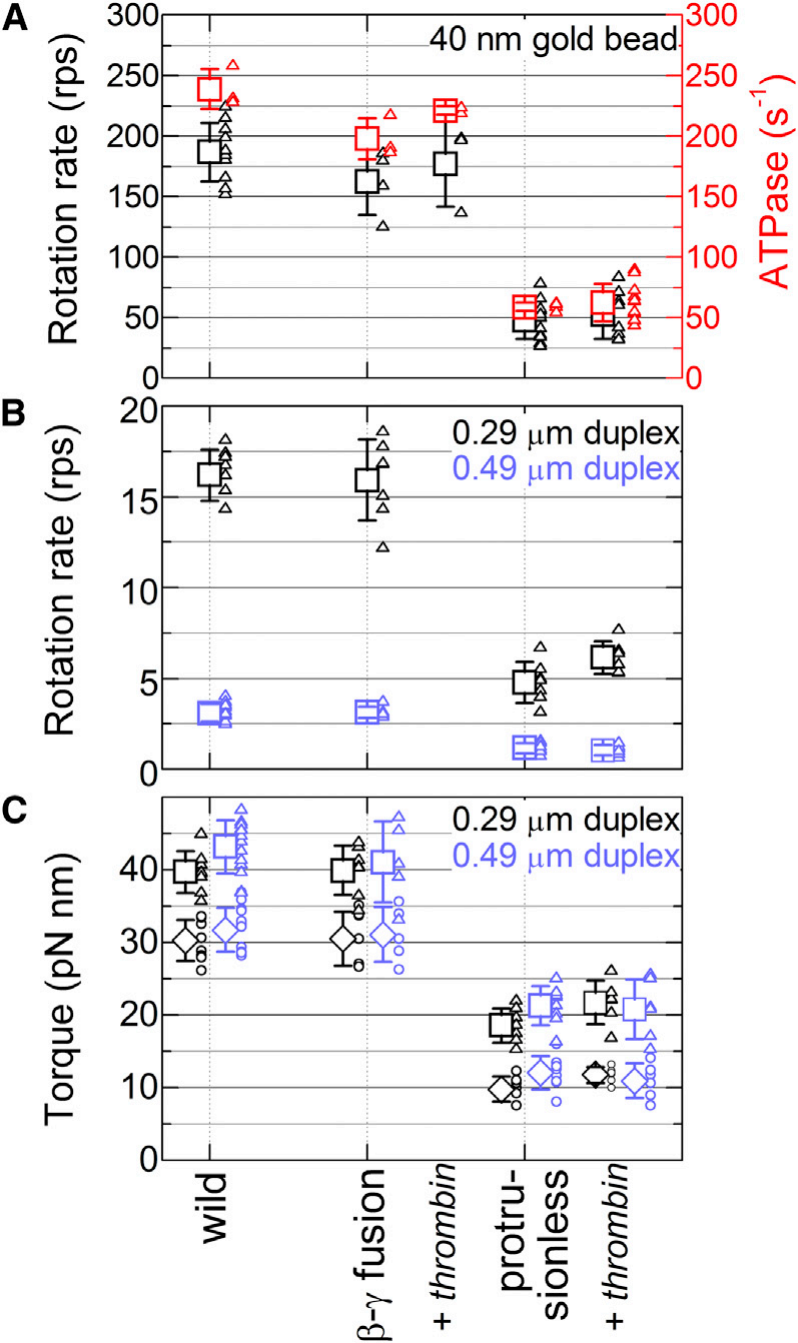
Summary of rotation and ATP hydrolysis activities at 2 mM MgATP. Small symbols are individual data and large symbols their average with error bars showing SD. (*A*) Time-averaged rotary speed for 40-nm gold beads (*black*), and hydrolysis activity in the presence of LDAO (*red*). (*B*) Time-averaged rotary speed of duplexes of polystyrene beads of indicated diameters. (*C*) Torque estimated from time-averaged rotary speeds of polystyrene bead duplexes (*diamonds*). Torque values estimated from the instantaneous rotary speed in consecutive 120° steps (Fig. 6) are also shown (*squares*). To see this figure in color, go online.

**Figure 5:**
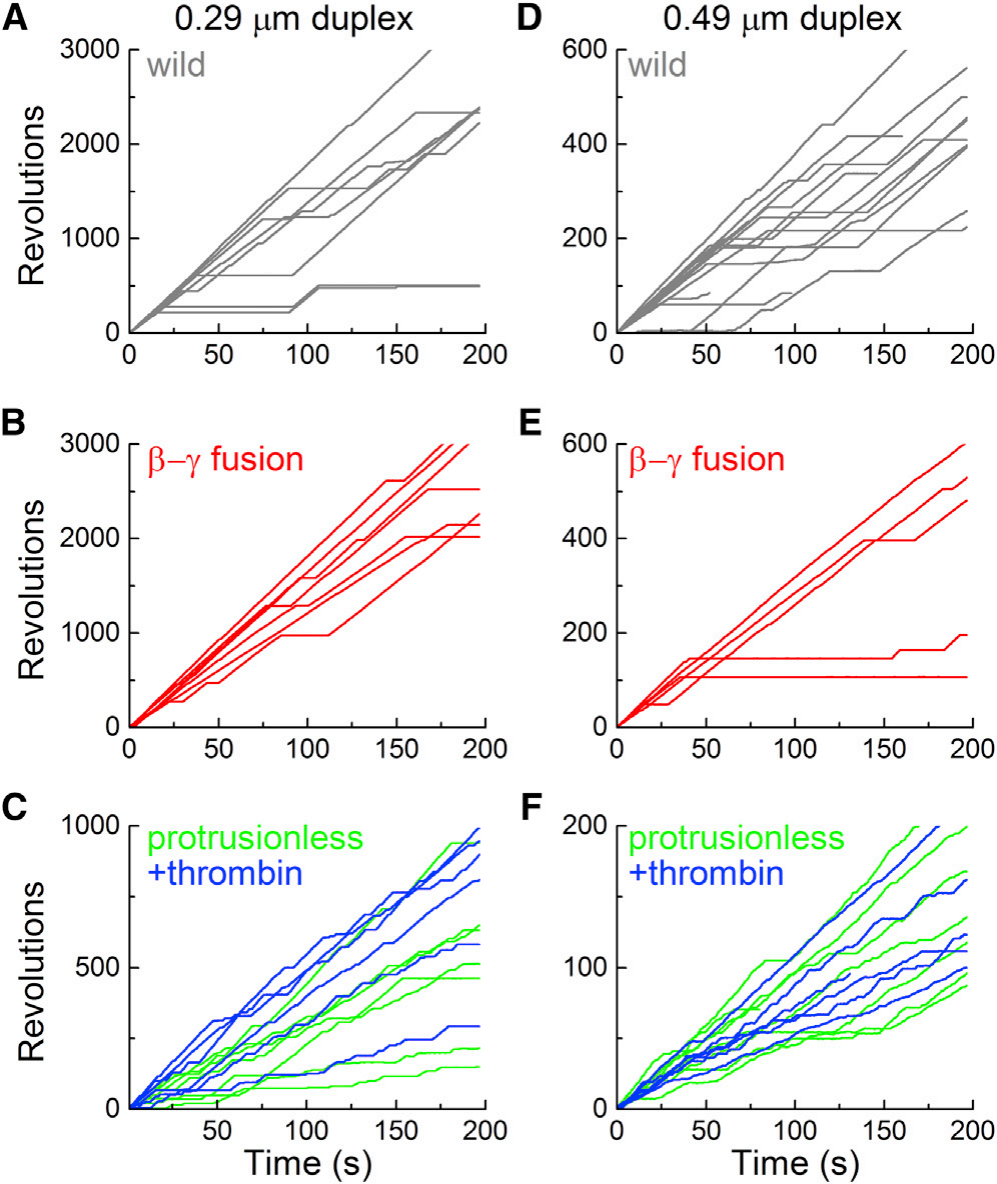
Time courses of polystyrene bead rotation at 2 mM MgATP. (*A*–*C*) Rotation of 0.29-*μ*m bead duplexes attached to the WT (*A*), the *β*-*γ* fusion (*B*), and the protrusionless mutant (*C*). Note the difference in the vertical scales. (*D*–*F*) 0.49-*μ*m bead duplexes. Time-averaged rotary speeds were estimated on uninterrupted portions (see text for the protrusionless mutant) and are plotted in Fig. 4*B* as small dots. To see this figure in color, go online.

WT TF_1_ attains its highest rotary speed when the probe attached to the rotor is smaller than 100 nm, implying that the hydrodynamic friction against a 40-nm gold bead should be negligible (27). At 2 mM ATP, which is saturating for the WT, the protrusionless TF_1_ rotated a 40-nm gold bead in the correct direction (counterclockwise when viewed from above) at a time-averaged speed of 48 ± 15 rps (estimated over >50 consecutive revolutions not interrupted by an obvious pause; mean over 12 beads; all errors shown in this work are standard deviations) and 52 ± 20 rps (8 beads) after thrombin treatment, both about one-quarter of the speed of the WT or the *β*-*γ* fusion (Figs. 3 and 4). The primary reason for the slower rotation seems to be brief dwells that occur mostly at 120° intervals (Fig. 3 *B*). The WT TF_1_ also exhibits dwells separated by 120° where ATP hydrolysis and phosphate release take place, the dwell positions being 80–90° behind the ATP-waiting angles (12). We have not confirmed whether the same reactions are involved in the dwells of the protrusionless TF_1_, but the dwells of the mutant were longer, suggesting slower hydrolysis kinetics in the protrusionless construct (Fig. 3 *B*). In addition to the brief dwells, the protrusionless mutant showed frequent pauses of the order of 1–10 s (Fig. 3 *A*). In the presence of 0.3% (w/v) LDAO, which would relieve F_1_ of the MgADP-inhibition (28), pauses longer than 1 s occurred 8 times in 640 s compared to 49 times in 590 s in the absence of LDAO (data not shown), suggesting that the pauses represent the inhibited states. The WT also fall in the Mg-ADP inhibited states, but the pauses are much longer and less frequent (29) and thus do not show up on the short timescale (see Fig. 5 below). We did not quantify the number of rotating gold beads, because judging rotation was more difficult with the protrusionless mutant than with the WT. Observation and analysis of rotation requires the bead center to be sufficiently off the rotation axis, but the mutant lacks the globular protrusion.

Commensurate with the slower rotation speed, the ATP hydrolysis activity of the protrusionless TF_1_ was severalfold lower than the WT. Because we did not remove the bound nucleotides completely, we compared the hydrolysis activity in the presence of 0.3% (w/v) LDAO. With LDAO, the hydrolysis rate did not change with time and amounted to 59 ± 3 s^−1^ (*n* = 4). This activity is about one-fourth of the WT activity (Fig. 4 *A*), and lower than three times the unloaded rotation rate expected for the three ATP per turn scenario. Presumably, part of the mutant protein had lost the integrity of the subunit assembly. The WT sample may also be partially inactive. As with gold bead rotation, the hydrolysis activity of the mutant did not change by thrombin treatment and was 63 ± 16 s^−1^ (*n* = 11).

### Torque generation

The torque needed to rotate the 40-nm gold bead is negligible. The frictional load would be only 0.6 pN nm for a rotation radius of 20 nm even at 200 rps. To see if the protrusionless mutant can generate a sizable torque, we attached a duplex of 0.29- or 0.49-*μ*m beads to the rotor tip. The protrusionless mutant was able to rotate both duplexes continuously, all counterclockwise. Compared to the WT, the mutant rotation was slower and interrupted with frequent pauses lasting one to tens of seconds (Fig. 5), as with the gold bead (Fig. 3). On the timescale of Fig. 5, pauses in the WT rotation due to the MgADP inhibition (29) are clearly discerned, but these were far less frequent compared to the mutant.

Rotating a submicron bead duplex requires a sizable torque, which mutants with a severely truncated rotor such as ΔC40 or ΔN14C36 could not provide. Time-averaged rotation speed of a bead duplex is a conservative measure of the torque the motor generates. The protrusionless mutant with a duplex of 0.29-*μ*m beads that lay nearly parallel to the glass surface (both beads focused nearly simultaneously) made 30 or more consecutive revolutions at 4.8 ± 1.1 rps (fastest portions, including pauses, of 7 duplexes) or 6.1 ± 0.9 rps (*n* = 6) after thrombin treatment, compared to 16.2 ± 1.4 rps (*n* = 8) for the WT or 15.9 ± 2.2 rps (*n* = 7) for the *β*-*γ* fusion (Fig. 4 *B*). For duplexes of 0.49-*μ*m beads, the mutant gave 1.1 ± 0.3 rps (14 duplexes, each for 20 or more consecutive revolutions, data for +/− thrombin combined), whereas the WT gave 3.1 ± 0.5 rps (*n* = 14). Thus, the protrusionless mutant generates at least 30–40% of the WT torque. For each duplex we estimated its hydrodynamic friction coefficient from Eq. 2, taking into account slight variations of the radius of revolution of the outer bead. We then calculated each torque by multiplying the time-averaged rotation speed by the friction coefficient (Eq. 1). The results, plotted in Fig. 4 *C*, averaged 10.7 ± 1.8 pN nm for the protrusionless mutant with a 0.29-*μ*m duplex (+/− thrombin) or 11.6 ± 2.3 pN nm with a 0.49-*μ*m duplex (+/− thrombin). The WT torque was 30.2 ± 2.8 pN nm (0.29 *μ*m) or 31.7 ± 3.0 pN nm (0.49 *μ*m). Again, the torque of the protrusionless mutant amounts to >30% of the WT torque. Note that these values are lower bounds because any stumbling and pauses in the consecutive rotation records were not omitted in the analysis.

**Figure 6:**
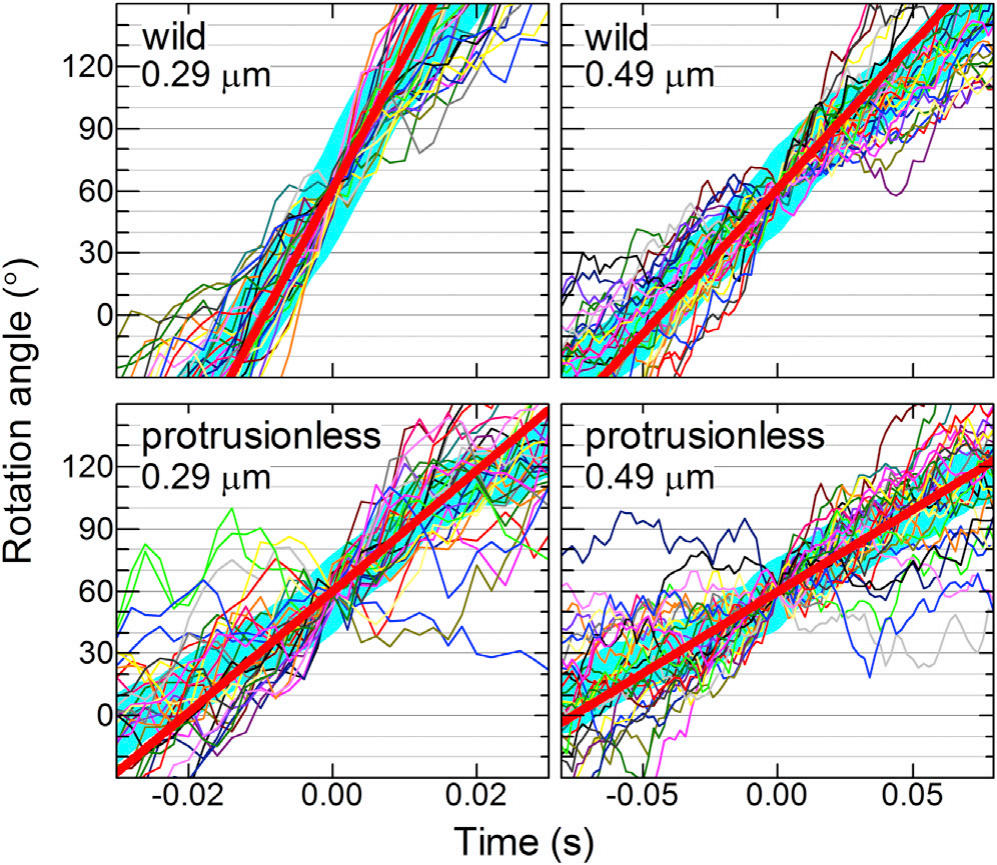
Torque estimation from 120° portions of a rotation record. Thin colored curves show 30 consecutive 120° steps overlaid on top of each other, thick cyan lines representing their average. Individual 120° steps in a continuous record were shifted vertically by a multiple of 120° to obtain the overlap. Time zero for each step was assigned by eye to the data point closest to 60°. Straight red lines indicate linear fit to the cyan curve between 30° and 90°. The slope of the red line, the angular velocity *ω* in rad s^−1^, gives the torque *N* (Eq. 1). To see this figure in color, go online.

A closer look at the rotation records indicated that the bead duplexes tended to stumble at angles separated by 120°, although irregularities are also seen at other angles. To eliminate the contribution of the short pauses at 120° intervals, we extracted 30 consecutive 120° portions from a rotation record and overlaid them on each other (Fig. 6). Subsequent averaging (*thick cyan line*) yielded a smooth step record, from which we estimated the slope between 30° and 90°. The torque of the protrusionless mutant estimated from this slope (instantaneous rotation speed) was 20.0 ± 3.1 pN nm for 0.29-*μ*m duplexes (*n* = 13, +/− thrombin) and 21.1 ± 3.2 pN nm for 0.49-*μ*m duplexes (*n* = 14, +/− thrombin), whereas WT values were 39.7 ± 2.9 pN nm for 0.29-*μ*m duplexes (*n* = 8) and 43.2 ± 3.6 pN nm for 0.49-*μ*m duplexes (*n* = 14), all somewhat higher than the torque estimated from time-averaged speeds (Fig. 4 *C*). We think that the torque values from the instantaneous speed, in which the short pauses were discounted, better represent the actual torque the mutant generates. The torque of the protrusionless mutant is likely ∼50% of the WT torque. Note that in these torque estimates we ignore the higher effective viscosity near a surface (24,26,30), and thus the absolute torque values are somewhat underestimated.

When we infused the same concentration of protein into an observation chamber, the bead density was lower with the protrusionless mutant than with the WT. In Table 1 we compare the numbers of beads found in six chambers each examined over 18 fields of view after infusing 5 nM of the protrusionless mutant or 1 nM of the WT. Despite the fivefold difference in the applied concentration, the number of beads attached to the mutant was only 70% larger than the WT. Probable reasons, among others, are difficulty in binding to the small rotor protrusion and surface denaturation of the mutant. The percentage of rotating bead duplexes was also smaller for the mutant than for the WT.

## Discussion

No residue-specific interactions are essential for finite torque generation (up to half the WT torque) in the F_1_ motor, at least in the F_1_ of thermophilic origin (TF_1_). Unloaded rotary speed also amounts to ∼50 rps, a quarter of the WT value, in the protrusionless mutant (this study) and ΔN50 where the rotor axle is vertically sliced in half (the entire N-terminal helix, *yellow* and *light yellow residues* in Fig. 1, *A* and *B*, was deleted in (17)). The acceleration from ∼1 rps in the axle-less (18) or rotorless (19) mutants must be due to feedback from the rotor to the catalytic stator, and this *γ*-dictated control is effective, though not perfect, without residue-specific interactions. Thus, F_1_ attains significant speed and torque without relying upon charge-charge interactions including salt bridges, interresidue hydrogen bonds, or steric effects on the atomic scale. The basic motor function rests on coarse steric interactions where the rotor’s shape and size alone at much lower than atomic resolution matter: any rod-shaped object that grossly mimics the coiled-coil axle of the *γ* subunit and that can be threaded through the stator cavity would serve as a rotor. It does not have to fill the cavity completely, because a counterpart of the coiled coil, the N-terminal helix, is entirely dispensable (whether a single *α*-helix suffices for the rotor function remains to be tested). It does not need a globular protrusion. It does not have to be as long as the native axle. Here we note, however, that the truncation of the C-terminal helix, the longer of the coiled-coil helices, impaired the unloaded speed rather severely: ΔC17 rotates at ∼22 rps and ΔC21 at ∼8 rps (18), although the torque of ΔC17 is ∼80% and ΔC21 ∼50% of the WT torque (31). Control of the progress of catalysis seems to require a longer rod.

To confer torque to, and to sense feedback from, a rotor solely through coarse steric interactions, the stator must maintain multiple contacts with the rotor. Through the contacts the stator keeps pushing/pulling the rotor at all rotary angles except for the ATP-waiting and hydrolysis/Pi-release dwells, and the rotor somehow mechanically promotes the catalysis, which is extremely slow in the absence of the rotor (19). How such mechanical coupling is achieved is not yet clear, despite the many crystal structures, mostly of MF_1_, solved so far. In these structures, the *γ* rotor interacts with the cylindrical *α*_3_*β*_3_ stator in two regions, the orifice region and the bottom of the central cavity of the stator (Fig. 1 *A*). TF_1_ is homologous to MF_1_ and its structure resembles the MF_1_ structure (32).

The very bottom of the stator cavity forms a hydrophobic sleeve, which would act as a molecular bearing for axle pivoting (8). Contacts with the sleeve are mostly dispensable for full torque generation: deletion of 12 C-terminal residues of *γ* in *E. coli* F_1_ (33) or 14 residues in TF_1_ (31) did not diminish the WT torque. Above the sleeve but still in the bottom region, MF_1_ *γ*-Arg-252 (corresponding to TF_1_ *γ*-Arg-264), *γ*-Arg-254 (TF_1_ *γ*-Arg-266), and *γ*-Gln-255 (TF_1_ *γ*-Gln-267) participate in a “catch” interaction involving hydrogen bonds and salt bridges with *β*_E_ (8). All bottom interactions, including these apparently conserved interactions, are dispensable for finite torque generation, because deleting the bottom half of the TF_1_ *γ* axle (ΔN11C32 or ΔN7C29) reduced the torque only by about one half (18). As regards the feedback signaling to the catalytic sites, the bottom interactions including the top of the sleeve are important as noted above. Presumably, a lever that spans the orifice and the bottom and thus could react against *β* bending is needed for efficient coordination among the three sites.

For basic torque generation, in contrast to the control of catalysis, coarse steric effects in the orifice region appear sufficient. Looking through sections perpendicular to the rotation axis in the crystal structures, however, we note that the rotor coiled coil is not contacted by the stator from all directions. In each section, there seems to be sufficient room for free rotation when the dispensable N-terminal helix is removed. Slippage must be prevented for torque generation. One possibility is that the stator subunits close in on the slim rotor during rotation. Or, the rotor, even if half sliced, is sufficiently rigid to serve as a lever encompassing all sections: a rigid lever does not necessarily require extensive contacts if proper contacts exist that transmit forces in correct directions. Maintaining a small number of proper contacts at all rotary angles, however, seems not easy, particularly because corrugations on the atomic scale are immaterial. Structures representing different stages of rotation are awaited for full explanation of the torque generation mechanism.

Theoretical simulations based on coarse-grained F_1_ models where the interactions between the *γ* rotor and the *α*_3_*β*_3_ stator involve steric repulsion alone have shown *γ* rotation in the correct direction (34,35). The successful reproductions of rotation, although the stator conformational changes were guided rather than spontaneous, are apparently in harmony with our results indicating that the basic torque generation relies solely on coarse steric interactions. Another coarse-grained model where electrostatic interactions are introduced in addition to steric repulsions also reproduced several aspects of rotation, stressing the importance of electrostatic terms (36). Our results argue against the importance, but this notion applies only to the basal torque and not to the full torque of the WT. In a recent study (37) the angular profile of the velocity of *γ* rotation in *E. coli* F_1_ has been measured with a tiny probe that would not be impeded significantly by the medium viscosity. The profile correlates with the theoretical torque profile based on steric repulsion alone (35). Assuming that the measured velocity is proportional to the motor torque, i.e., that the friction is somehow independent of the rotary angle, the authors (37) take the correlation as evidence for steric repulsion being the major determinant of the torque (of untruncated F_1_).

Studies that point to the importance of specific interactions also exist, particularly for the orifice region. There, an extensive catch interaction exists between *γ* and *β*_(TP)_ (8): MF_1_ *γ*-Ala-80 (*γ*-Ala-90 in TF_1_), *γ*-Lys-87 (*γ*-Arg-97), and *γ*-Lys-90 (*γ*-Tyr-100) in a *γ* short helix form hydrogen bonds to residues in a conserved acidic DELSEED (*β*394–400) motif in MF_1_ *β* or the DELSDED motif of TF_1_ *β*390–396. Deletion of this short *γ* helix has not been tested, although removal of all charges on the DELSDED motif of TF_1_ (38) or even partial deletion of this motif (39) did not change the torque appreciably. Here, we show that the protrusionless construct without this short helix generates at least one-third, and likely one-half, of the WT torque. MF_1_ *γ*-Met-23 is conserved among species and its mutation to a positively charged residue has been shown to affect the activity in *E. coli* (40) and *Rhodobacter capsulatus* (41). Here, we replaced this residue (TF_1_ *γ*-Met-24) with hydrophobic Ile, but we have already shown that this residue is dispensable (17). We thus conclude that TF_1_ can generate a sizable (∼half of WT) torque without a residue-specific interaction.

Our results point to the possibility that F_1_ evolved from a low speed, low power motor with a poorly (or arbitrarily) designed rotor. To synthesize ATP by reverse rotation, the torque must be high, because the energy input for the synthesis is the mechanical work against the torque F_1_ generates, i.e., the torque times 2*π*/3 radian (120°). In cells the free energy needed for ATP synthesis is 80–100 pN nm (50–60 kJ mol^−1^), and thus F_1_ capable of synthesis must generate at least 40 pN nm of torque. The present WT TF_1_ meets this condition. Likely, F_1_ started to synthesize ATP when it had evolved to generate this much torque. Or, in early times the ATP concentration in cells might have been low or the ADP concentration high such that the free energy required for synthesis was also low. The demonstration of ATP synthesis by a mutant generating only ∼half the WT torque (39) might point to this possibility.

## Figures and Tables

**Table 1:** Number of 0.29-*μ*m beads observed on surfaces with the WT or protrusionless TF_1_

	Rotating duplexes	Fluct. duplexes	Stuck duplexes	Higher aggre.	Single beads	Total
Wild (1 nM)						
Ch 1	38	191	109	374	958	1670
Ch 2	31	132	73	149	909	1294
Ch 3	27	140	70	130	700	1067
Ch 4	31	144	62	163	622	1022
Ch 5	29	65	31	51	460	636
Ch 6	19	80	48	53	552	752
Mean	29.2	125.3	65.5	153.3	700.2	1073.5
SD	6.2	46.1	26.4	118.2	197.9	374.7
%Total	2.7%	11.7%	6.1%	14.3%	65.2%	100%
Protrusionless (5 nM)						
Ch 1	16	288	281	616	1726	2927
Ch 2	6	135	97	159	949	1346
Ch 3	14	108	138	142	764	1166
Ch 4	10	141	174	247	977	1549
Ch 5	14	141	162	186	1362	1865
Ch 6	11	129	125	113	1038	1416
Mean	11.8	157.0	162.8	243.8	1136.0	1711.5
SD	3.6	65.3	64.0	187.9	348.5	639.7
%Total	0.7%	9.2%	9.5%	14.3%	66.4%	100%

For each chamber (Ch 1 to 6), randomly selected 18 fields of view (31 × 31 *μ*m^2^) were examined for 12 s at 2 mM ATP. Some beads stopped or started rotation during the observation period, and these were counted as rotating. Fluctuating and stuck duplexes were distinguished by eye. Higher aggregates and singles were counted as such irrespective of whether moving or not. Without TF_1_, 13 beads were found per chamber.
